# Progress in Diseases of the Heart

**Published:** 1901-02-09

**Authors:** 


### Progress in Diseases of the Heart.

**Physiology.**—Evidence in favour of the heart having the power of enlarging its chambers by means of a true muscular expansion movement has been brought forward by Stacey Wilson.[1]

He mentions that in the cases in his experience of compensated mitral regurgitation the left auricle shared in hardly any respect in the hypertrophy and dilatation of the ventricle. This he holds reveals the *vis a fronte* in the shape of the suction-power of the ventricle.

He also shows a number of tracings taken from cases of anæmic dilatation, mitral regurgitation, and the latter with tricuspid regurgitation, and argues that the curve produced by the expansion of the ventricle shows an active effort on the part of the muscle.

In mitral stenosis the ventricle seems to have increased rather than diminished work to perform; as shown by slight hypertrophy and louder first sound. This he holds to be due to the effort to empty itself as completely as possible that its expansion later shall exercise as much suction as possible.

His tracings from such cases show at the commencement of diastole a small wave, which he calls the "suction recoil wave," and which he considers is caused by the blood in the auricle still pouring into the relaxed ventricular cavity after the active expansion of that cavity has taken place; nominally, the auricular contents should have all been emptied into the ventricle, but, stenosis being present, some is left to fall into the ventricle while it is relaxed. In the so-called reduplication of the second sound in these cases, the "third sound" is coincident with the suction recoil wave, and is produced after the mitral valve is opened and is caused by the flow of the remaining blood into the ventricle; after that the auriculo-ventricular valve is left free to move, the latter being carried back with a sound against the ventricular wall.

The "mid-systolic" murmur has a similar explanation it replaces the "third" sound.

In aortic regurgitation there is no expansion wave; active expansion would be harmful, and thus the beat is changed so as to produce the minimum of the diastolic expansion.

Professor Potain[2] claims that the interpretation of Dickinson and Chauveau that the auriculo-ventricular valve is rendered temporarily insufficient by means of the over-contraction of the musculi papillares, and so allows the production of the intersystolic bruit, is not a correct interpretation of the phenomenon. He considers it due to the action of the auricle.

**Diagnosis.**—O'Carroll[3] throws some doubt upon the correctness in accepting Skoda's interpretation of the "Accentuation of the second sound in the pulmonary area." He brings forward the notes of three cases occurring in his practice, in which the accentuation of the second sound in this area disappeared coincidently with the development of aortic regurgitation. He reasons that owing to the anatomical position, the aortic second sound is heard more plainly in the pulmonary area, and that the accentuation "is produced at the aortic orifice as the result of increased amplitude of the recoil wave in the systemic arteries," this being produced by a diminished quantity of blood being thrown into them by a vigorous ventricle (mitral disease), or by diminished vaso-motor tonus, as in children and neurotic adults.

With this view, he urges that the physician is able to have evidence of "the efficiency of the left ventricle, the quantity of blood thrown into the aorta at systole, and the resiliency of the arterial walls. Also, the intensity of the second sound in pulmonary area is no clue to the compensatory activity of the right heart."

Stacey Wilson[4] points out that in mitral regurgitation the murmur may be heard over the spines of the dorsal and lumbar vertebræ, and even over the pelvic bones. He holds that this conduction is due to the anatomical relationship of the left auricle and left ventricle to the bodies of the vertebræ. When dilated the heart pushes away the lungs, and comes into immediate contact with the vertebral bodies and posterior chest wall.

He states that the conduction of a murmur down the vertebral column is evidence of a considerable amount of mitral regurgitation, and the extent of its conduction may be regarded as an index of the amount of regurgitation. Also, it is evidence of dilatation of the left auricle and ventricle. A diagnosis can be made between mitral and tricuspid regurgitant murmurs as the mitral are conducted much more clearly. Aortic murmurs are heard over the upper vertebræ more than the lower, the mitral murmur being heard best over the fourth and fifth dorsal spines, disappearing the higher above this you go.

An aneurysm of the descending arch gives rise to a systolic murmur in the interscapular area. Consolidation of the lung, or the presence of tumours, may cause normal heart sounds to be heard more plainly than normal, and this will apply to murmurs also; but the presence of other symptoms would be the guide. Pulmo-cardiac murmurs are heard sometimes very clearly posteriorly, but may be heard over the lower lobe, below the angle of the scapula, but not over the vertebræ, and are not best heard in the mid-interscapular region. With regard to prognosis, if the sound is not conducted down the vertebræ the damage to the valve is slight; but if it is conducted down far, then the damage is great. Loudness is suggestive of dilatation.

Norman Moore[5] states that careful auscultation at the back will differentiate murmurs in the same heart. The mitral murmurs being loudest at the angle of the left scapula, the aortic murmur will often be well marked in the right supraspinous fossa. Tracing down from the latter to the former areas, we find that in mitral regurgitation with aortic constriction, the murmur will die away and suddenly become loud again at the angle of the scapula.

Ewart[6] lays emphasis upon the "lower dorsal dull patch" in the diagnosis of pericardial effusion as opposed to enlarged heart. For it is sharply defined and does not fit any other diagnosis. The dulness is almost median in position below the tenth rib, and may extend into the right chest for one or two inches. This cannot be produced by enlargement of the heart, the patch of dulness being situated below the level of the floor of the pericardium.

Michell Clarke[7] reports a case of varicose aneurysm between the aorta and pulmonary artery, with a gradual onset. For this rare condition he gives the following diagnostic points:—Shortness of breath, palpitation, dropsy, the peculiar murmur and thrill heard and felt at the base to the left of the sternum, and possibly over the right ventricle; enlargement of the heart and especially dilatation of the right ventricle; rapid development of general dropsy, with scanty excretion of urine; absence of marked cyanosis and orthopnœa; moderate increase in the rate of the pulse, which may be, but is not always of the regurgitant type; great engorgement of liver, and possibly a sense of well-being relatively to the serious condition present.

**Angina Pectoris.**—Dr. Theodor Schott[8] writes a paper in which, after a review of the pathology and therapy of angina pectoris, he upholds the Parry-Stokes theory of its causation; viz., that the attack is due to a further reduction of the muscular energy of the already enfeebled heart. Parry believes the symptoms to be due to an accumulation of blood in the cardiac cavities. Schott maintains that the heartsounds become feebler during the attacks in some cases, in others mumurs or a galloping rhythm; that also the heart is distended and may show dilatation of all its cavities, the left side beginning first. This may be proved by diagram—as in a case of his.

With regard to treatment he has used heat and hot friction with great relief of the pains. He speaks to the value of nitro-glycerin used under medical supervision, morphine, iodide of sodium in milk, and well ordered bath treatment. All this treatment he regar's as means of lowering the pressure of the blood, and giving the enfeebled heart less work to do; the bath and tonic treatment tending to strengthen the heart muscle.

[1] Brit. Med. Jour., Sept. 29. [2] La Semaine Médicale, Nov. 14. [3] Dublin Jour. Med. Sci., September. [4] Birmingham Med. Rev., October. [5] Lancet Oct. 20. [6] Lancet, Nov. 3. [7] Brit. Med. Jour., Dec. 15. [8] Lancet, Sept. 8'

